# Role of noble metal-coated catheters for short-term urinary catheterization of adults: a meta-analysis

**DOI:** 10.1371/journal.pone.0233215

**Published:** 2020-06-10

**Authors:** Yan Sun, Ping Ren, Xuan Long

**Affiliations:** 1 Department of Encephalopathy Rehabilitation, Zaozhuang Traditional Chinese Medicine Hospital, Zaozhuang, Shandong, P.R. China; 2 Drug Distribution Center, Zaozhuang Traditional Chinese Medicine Hospital, Zaozhuang, Shandong, P.R. China; 3 Department of Infectious Diseases, Zaozhuang Municipal Hospital, Zaozhuang Shandong, P.R. China; University of York, UNITED KINGDOM

## Abstract

**Objective:**

To evaluate the efficacy of noble-metal coated catheters in reducing catheter-associated urinary tract infections (CAUTI) in adult patients requiring short term catheterization.

**Methods:**

An electronic literature search of PubMed, BioMed Central, Embase, Scopus, CENTRAL (Cochrane Central Register of Controlled Trials) and Google scholar was carried out from inception to 10th October 2019. Any prospective study or randomized controlled trial (RCT) on adult patients comparing noble-metal coated urinary catheters with any standard catheter and evaluating the incidence of CAUTI or bacteriuria was included.

**Results:**

A total of 13 studies were included in the systematic review. 12 were RCTs and one was a prospective cross-over trial. Catheters employed in the study group were grouped into two sub-groups: Silver alloy coated or Noble metal alloy-coated (Gold, Silver, and Palladium) catheters. Bacteriuria was the most commonly studied outcome variable across trials. Meta-analysis indicated that silver alloy-coated catheters (RR 0.63, 95%CI 0.44–0.90, P = 0.01; I2 = 72%) and noble metal alloy catheters (RR 0.58, 95%CI 0.41–0.81, P = 0.001; I2 = 0%) significantly reduce the risk of bacteriuria. Sub-group analysis based on the duration of catheterization demonstrated that silver alloy catheters reduce the risk of bacteriuria with >1week of catheterization (RR 0.46, 95%CI 0.26–0.81, P = 0.007; I2 = 63%). Symptomatic CAUTI was evaluated only in four studies with variable results. The quality of the included studies was not high.

**Conclusions:**

Our review indicates that bacteriuria may be reduced with the use of noble metal-coated catheters during short-term catheterization of adults, however, the quality of evidence is not high. It is not clear if these catheters reduce the risk of symptomatic CAUTI. Further homogenous RCTs are needed to provide clarity.

## Introduction

Urinary tract infections (UTIs) are amongst the most common nosocomial infections with indwelling catheters contributing to an estimated 80% of these disorders [1,2]. Catheter-associated UTIs (CAUTIs) can lead to significant morbidity with prolonged hospital stays and escalation in treatment costs in both high-income and low-income countries. Umscheid et al in a review have demonstrated that approximately 65–70% of CAUTIs may be preventable with current infection-control strategies. [3]

In recent times, nursing personnel are being increasingly involved in the prevention of hospital-acquired infections. A number of nurse-led protocols, with greater participation of nurses in monitoring and timely removal of urinary catheters, have been reported [4,5]. Nurses are not only involved in daily catheter care but also in the selection of appropriate catheters to reduce the incidence of CAUTIs [6]. While standard uncoated catheters are frequently employed by health-care providers, several anti-microbial and anti-septic coated catheters are available which may reduce the risk of CAUTIs. Catheters have been coated with silver alloy, noble metal alloy, chlorhexidine and nitrofurazone with the sole objective of reducing the risk of CAUTIs [7,8].

The anti-septic properties of noble metals consisting of gold, silver, palladium have been utilized in numerous fields of medicine [9]. Thin-coatings of silver in the form of silver oxide or silver alloy and noble metal alloy (Gold, silver, and palladium) have been applied on urinary catheters to reduce bacterial adherence and theoretically decrease the risk of CAUTIs. Several clinical trials have compared the use of these noble metal-coated catheters with standard catheters but with conflicting results [10,11]. In a Cochrane review by Lam et al, the authors had evaluated evidence on the efficacy of such noble-metal coated catheters in 2014 [8]. Since then many new trials have been published and there is a need for updated evidence on this subject, to provide guidelines to health-care providers [12–15]. Hence, the primary objective of this review was to perform a systematic literature search and conduct a meta-analysis to answer the clinical question: Does the use of noble-metal coated urinary catheters for short-term catheterization of adults reduce the incidence of CAUTI?

## Materials and methods

### Criteria for study inclusion

The review was performed following the PRISMA statement (Preferred Reporting Items for Systematic Reviews and Meta-analyses) [16] and the Cochrane Handbook for Systematic Reviews of Intervention [17]. Following the PICOS (Population, Intervention, Comparison, Outcome, and Study design) outline, we included any prospective study or RCT carried out on a *Population* of adult patients (>18years) requiring short-term urinary tract catheterization for any reason. *The intervention* was to be the use of a noble-metal coated urinary catheter, *Compared* with any standard catheter not coated with noble-metal or any antimicrobial agent. *The outcome* of the study was to be the incidence of CAUTI or bacteriuria. Short-term catheterization was preferably defined as the use of the catheter for ≤14 days. However, studies were also included if some patients were catheterized for >14 days but the mean duration of catheterization was ≤14 days. We excluded the following studies: 1. Studies using silver-oxide coated catheters (as these are no more manufactured) and utilising any other anti-microbial coated catheters 2. Conducted on patients requiring chronic/long-term catheterization 3. Utilizing supra-pubic catheter 4. Retrospective studies, case series, and case reports.

### Search strategy

A systematic literature search of various electronic databases including PubMed, BioMed Central, Embase, Scopus, CENTRAL (Cochrane Central Register of Controlled Trials) and Google scholar was carried. We searched all databases from their inception to 10^th^ October 2019. No restriction was placed on the language of publication. Two independent reviewers performed the literature search using the MeSH terms and free-text keywords. “Noble metal catheter”, “Silver”, “Silver alloy”, “Silver oxide”, “urinary catheter”, “Foleys catheter”, “infection”, and “urinary tract infection” were used in various combinations. We manually checked the reference lists of all included studies and review articles for any additional references. The literature search results were screened by their titles and abstracts by two independent reviewers for every database. Potentially relevant articles were then extracted and subsequently screened by their full text. Both the reviewers assessed individual studies based on inclusion criteria and resolved any disagreement, by discussion.

### Data extraction and risk of bias assessment

A data abstraction form was used by the reviewers to source data from the selected studies. Details of authors, publication year, inclusion/exclusion criteria, sample size, demographic data, types of catheters used, duration of catheterization, outcomes, and study results were extracted. The outcomes of interest were to assess the difference in the incidence of symptomatic/asymptomatic CAUTI and bacteriuria between the two study groups.

Two review authors independently assessed the risk of bias in included studies using the Cochrane Collaboration risk assessment tool for RCTs [18]. Every study was assessed on the following domains: random sequence generation, allocation concealment, blinding of participants and personnel, blinding of outcome assessment, incomplete outcome data, selective reporting, and other biases. Each domain was graded as high risk, low risk or unclear risk of bias and results presented pictorially in a *Risk if bias* figure.

### Statistical analysis

Anticipating methodological heterogeneity in the included studies, a random-effects model was preferred to calculate the pooled effect size. We summarized categorical data using the Mantel-Haenszel Risk Ratio (RR) with 95% confidence intervals (CI). Heterogeneity was calculated using the I^2^ statistic. I^2^ values of 25–50% represented low, values of 50–75% medium and more than 75% represented substantial heterogeneity. Sub-group analysis was conducted for different noble-metal coatings and different duration of catheterization (>1 week or <1week). A sensitivity analysis was carried out to assess the influence of each study on the pooled effect size. As the pooled outcome of each sub-group did not include more than 10 trials, we could not create a funnel plot to explore possible small study and publication biases. The software “Review Manager” (RevMan, version 5.3; Nordic Cochrane Centre [Cochrane Collaboration], Copenhagen, Denmark; 2014) was used for the meta-analysis.

## Results

**Fig 1:**
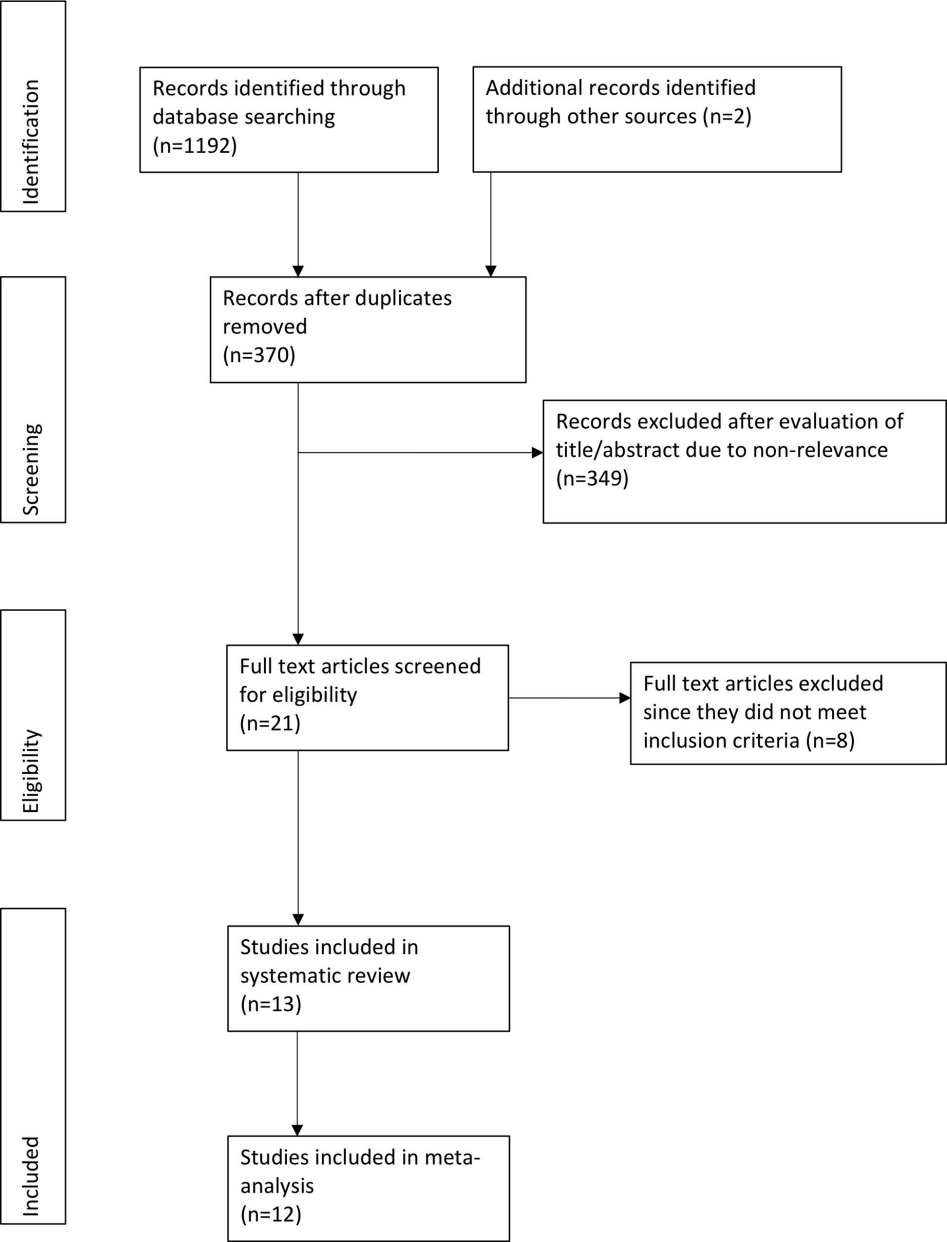
Study flow chart.

A comprehensive literature search revealed a total of 370 unique records (Fig 1). The search strategy for PubMed database is presented in S1 Table. A total of 21 articles were analyzed by their full-texts of which 8 were excluded[19–26]. Three employed silver-oxide catheters[19,21,22], two studies were only in abstract form [20,23], two studied the effects of long-term catheterization [25,26] while one study compared two different types of silver-coated catheters [24]. A total of 13 studies were included in the review [10–15,27–33]. Details of the included studies are presented in Table 1. One study [10] did not report data as the number of events (CAUTI or bacteriuria)/ total number of patients, and hence was not included in the meta-analysis. One paper had two sub-reports in the same article which were analyzed separately [30]. Except for one prospective cross-over trial [15], all were RCTs. There was a large variation in the sample size in the studies included in the meta-analysis, varying from 11 to 2144 participants per group. The largest sample size was, however, of the study not included in the meta-analysis with >13900 patients per group [10]. Different types of noble metal-coated, as well as standard catheters, were used across studies. Catheters employed in the study group were grouped into two sub-groups: Silver alloy coated or Noble metal alloy-coated (Gold, Silver, and Palladium) catheters. For the comparative arm, studies described the catheters as standard/regular catheter, standard silicone catheter, standard latex catheter, standard silicone latex catheter, polytetrafluoroethylene (PTFE) coated latex catheter, teflonised latex catheter, standard hydrogel coated catheter and standard hydrogel-coated latex catheter. The mean, median or total duration of catheterization was ≤14 days for all studies except one study where catheterization was done up to 22 days [12]. However, the authors reported outcomes of bacteriuria at frequent intervals from 2–22 days. Outcome data up to 14 days was extracted from this study.

**Table 1:** Characteristics of included studies.

Author/Year	Study Type	Study population	Sample size (n)		Male gender (n)		Study group catheter	Control group catheter	Duration of catheterization
			Study	Control	Study	Control			
Lundeberg et al [27]/ 1986	RCT*	NR	51	51	NR	NR	Silver coated catheter	Standard catheter	NA
Liedberg et al-1 [34]/ 1990	RCT	Patients requiring hemodynamic monitoring or post-operative drainage	30	60	NA	NA	Silver alloy catheters	Standard hydrogel coated or Standard non-coated catheter	5 days^#^
Liedberg et al-2 [28]/ 1990	RCT	Patients requiring hemodynamic monitoring or post-operative drainage	60	60	43	40	Silver alloy catheters	Teflonised latex catheter	6 days^#^
Verleyen et al-1 [30]/ 1999	RCT	Post radical prostatectomy patients	12	15	12	15	Silver alloy hydrogel catheters	Standard catheter (silicone)	14 days^#^
Verleyen et al-2 [30]/ 1999	RCT	Urological surgery	79	101	47	55	Silver alloy hydrogel catheters	Standard catheter (Latex)	5 days (median)
Karchmer et al [10]/ 2000	RCT	Patients requiring hemodynamic monitoring or post-operative drainage	13945	13933	NR	NR	Silver alloy hydrogel latex catheters	Standard hydrogel-coated latex catheter	NA
Thibon et al [31]/ 2000	RCT	ICU and neurosurgery patients	90	109	NA	NA	Silver alloy hydrogel catheters	Standard catheter (silicone)	Study: 5.8±2.5 days Control: 5.9±2.3 days (mean±SD)
Stenzelius et al [32]/2011	RCT	Orthopedic surgery patients	222	217	96	82	Gold, silver, and palladium coated latex catheter	Standard catheter (silicone)	2 days (mean)
Pickard et al [11]/ 2012	RCT	Patients requiring hemodynamic monitoring or post-operative drainage	2097	2144	778	815	Silver alloy hydrogel-coated latex catheter	Polytetrafluoroethylene (PTFE) coated latex catheter	≤14 days^#^
Fabrellas et al [14]/ 2013	RCT	Post-operative cardiac surgery patients	58	58	35	39	Gold, silver, and palladium coated catheter	Standard catheter (Latex)	4 days (mean)
Aljohi et al [13]/ 2016	RCT	ICU patients	30	30	14	16	Latex gold, silver, palladium and hydrogel layer coated catheter	Standard catheter (silicone latex)	3 days^#^
Stenzelius et al [15]/2016	Prospective cross-over	Stroke or acute neurological condition	151	171	84	82	Latex gold, silver, palladium and hydrogel layer coated catheter	Standard catheter (silicone)	Study: 8.8±11.1 Control: 9.1±8.2 (mean±SD)
Akcam et al [12]/ 2019	RCT	ICU patients	28	26	16	11	Silver-coated silicone catheters	Standard catheter (silicone)	Up to 22 days in both groups^@^
Ardehali et al [33]/ 2019	RCT*	NR	157	157	NR	NR	Gold, silver, and palladium coated catheter	Regular catheter	2–7 days^#^

*published as letter to editor ^#^total length of catheterization ^@^Data available for up to 14 days.

NR, Not reported; NA, Data not extractable (not available); SD, Standard Deviation; ICU, intensive care unit.

### Outcomes

Outcomes and their definitions varied across studies (Table 2). Bacteriuria was the most commonly studied outcome variable across trials. While majority studies defined bacteriuria as ≥10^5^ Colony-forming units (CFU)/ml, one study considered bacteriuria as ≥100 CFU/ml [27]. Combined clinical and microbiological criteria to define symptomatic CAUTI were used only in four studies [10,11,13,15], which too were variable.

**Table 2:** Outcomes of included studies.

Author/Year	Outcomes and definitions	Results
Lundeberg et al [27]/ 1986	Bacteriuria: ≥100 CFU/ml	Significant difference between study and control groups
Liedberg et al-1 [34]/ 1990	Bacteriuria: ≥10^5^ CFU/ml	Significant difference between study and control groups
Liedberg et al-2 [28]/ 1990	Bacteriuria: ≥10^5^ CFU/ml	Significant difference between study and control groups
Verleyen et al-1 [30]/ 1999	Bacteriuria: ≥10^5^ CFU/ml	Non-significant difference between study and control groups
Verleyen et al-2 [30]/ 1999	Bacteriuria: ≥10^5^ CFU/ml	Significant delay in the onset of bacteriuria with silver catheters
Karchmer et al [10]/ 2000	UTI: defined according to a pre-specified clinical and microbiological criteria Asymptomatic Bacteriuria: ≥10^5^ CFU/ml with no more than 2 species of organism and after >7days of indwelling catheter	A significant 21% reduction of risk of infection per 1000 patient-days in study group
Thibon et al [31]/ 2000	UTI: Bacteriuria of ≥10^5^ CFU/ml and >10 leucocytes/mm^3^	Non-significant difference between study and control groups
Stenzelius et al [32]/2011	Bacteriuria: ≥10^5^ CFU/ml	Significant difference between study and control groups
Pickard et al [11]/ 2012	Symptomatic UTI: presence of participant-reported symptoms of urinary tract infection and clinician prescription of antibiotic for a urinary tract infection at any time up to 6 weeks	Non-significant difference between study and control groups
	Microbiologically confirmed symptomatic UTI: symptomatic UTI and a positive urine culture	
	Symptomatic or asymptomatic bacteriuria: ≥10^5^ CFU/ml	
	Urethral discomfort with catheter	
Fabrellas et al [14]/ 2013	Bacteriuria: ≥10^5^ CFU/ml	Significant difference between study and control groups
Aljohi et al [13]/ 2016	UTI: based on definition from Center for Disease Control and Prevention (CDC) ‐ National Healthcare Safety Network criteria	Significant difference between study and control groups
	Catheter associated Asymptomatic Bacteriuria: urinary catheter within 48 h before the specimen collection, a positive urine culture of ≥10^3^ CFU/ml or ≥10^5^ CFU/ml with no more than two organisms, one laboratory evidence if <10^5^ CFU/ml	
	Asymptomatic Bacteriuria: ≥10^5^ CFU/ml with no more than 2 species of organism, no signs and symptoms of UTI, positive blood culture with at least one matching bacteria to the urine culture	
	Symptomatic Bacteriuria: same as above with symptoms of UTI	
Stenzelius et al [15]/2016	UTI: symptoms of the urinary tract (lower and/or upper) combined with the presence of bacterial growth in urine (>10^4^ CFU/ml), and prescription of urinary tract antimicrobial agent	Non-significant difference between study and control groups
Akcam et al [12]/ 2019	Bacteriuria: ≥10^5^ CFU/ml	Non-significant difference between study and control groups
Ardehali et al [33]/ 2019	Symptoms of UTI	Non-significant difference between study and control groups
	Bacteriuria: ≥10^5^ CFU/ml	

CFU, Colony forming units; UTI, urinary tract infection.

**Fig 2:**
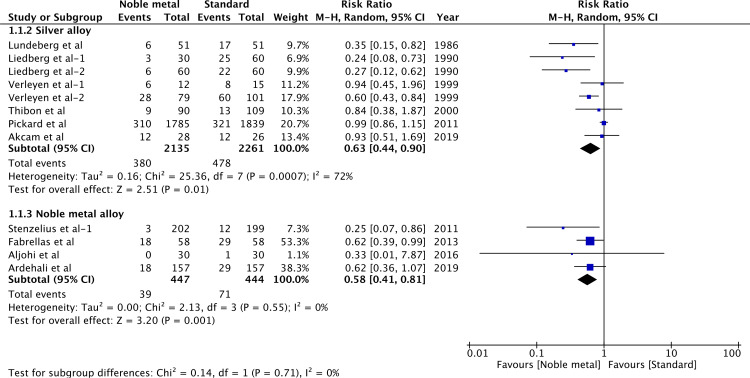
Forrest plot of bacteriuria.

Data of bacteriuria (till the last follow-up) was pooled for a meta-analysis based on the type of noble metal coating (Fig 2). Seven studies [11,12,27,28,30,31,34] with eight reports were pooled for silver alloy catheter vs standard catheter. Results indicated a significant decrease in the risk of bacteriuria with silver alloy-coated catheters (RR 0.63, 95%CI 0.44–0.90, P = 0.01; I^2^ = 72%). Similarly, when data of four trials [13,14,32,33] was pooled for comparing bacteriuria with noble metal alloy catheters vs standard catheters, there was a significantly reduced risk of bacteriuria with noble metal alloy catheters (RR 0.58, 95%CI 0.41–0.81, P = 0.001; I^2^ = 0%).

**Fig 3:**
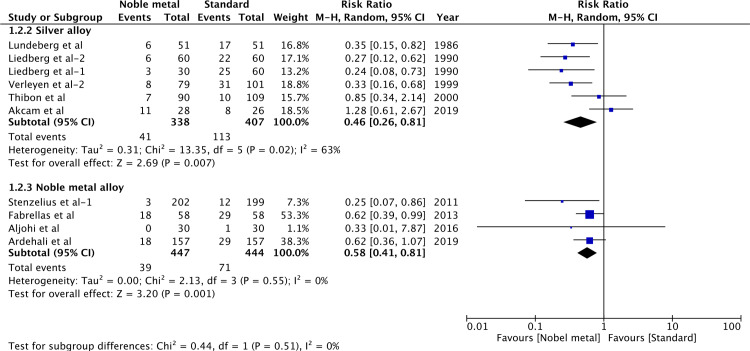
Forrest plot of bacteriuria(<1week of catheterization).

**Fig 4:**
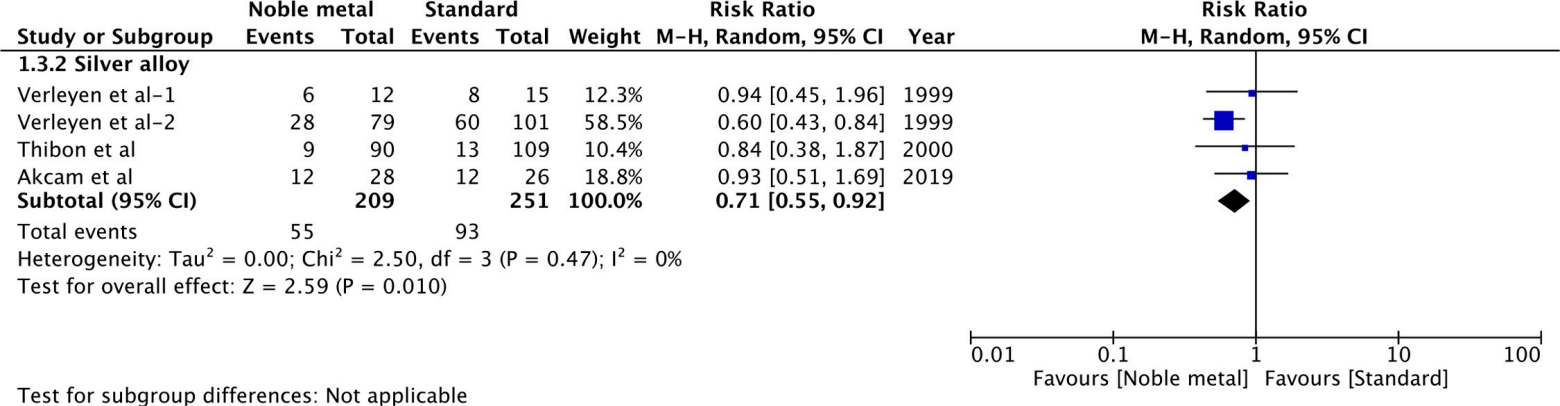
Forrest plot of bacteriuria(>1week of catheterization).

Data from studies reporting bacteriuria after catheterization of <1week and >1week were pooled separately. As all studies for noble metal alloy catheters reported data of <1week, the results were the same as prior analysis (Fig 3). The results were still significant for silver-alloy catheters (RR 0.46, 95%CI 0.26–0.81, P = 0.007; I^2^ = 63%) when used for a catheterization period of <1week (Fig 4). A total of three trials [11,12,30,31] with four reports were pooled for the incidence of bacteriuria after >1week of indwelling silver alloy catheter vs standard catheter. Our results indicated significant difference between the two groups (RR 0.71, 95%CI 0.55–0.92, P = 0.01; I^2^ = 0%) (Fig 4).

**Fig 5:**
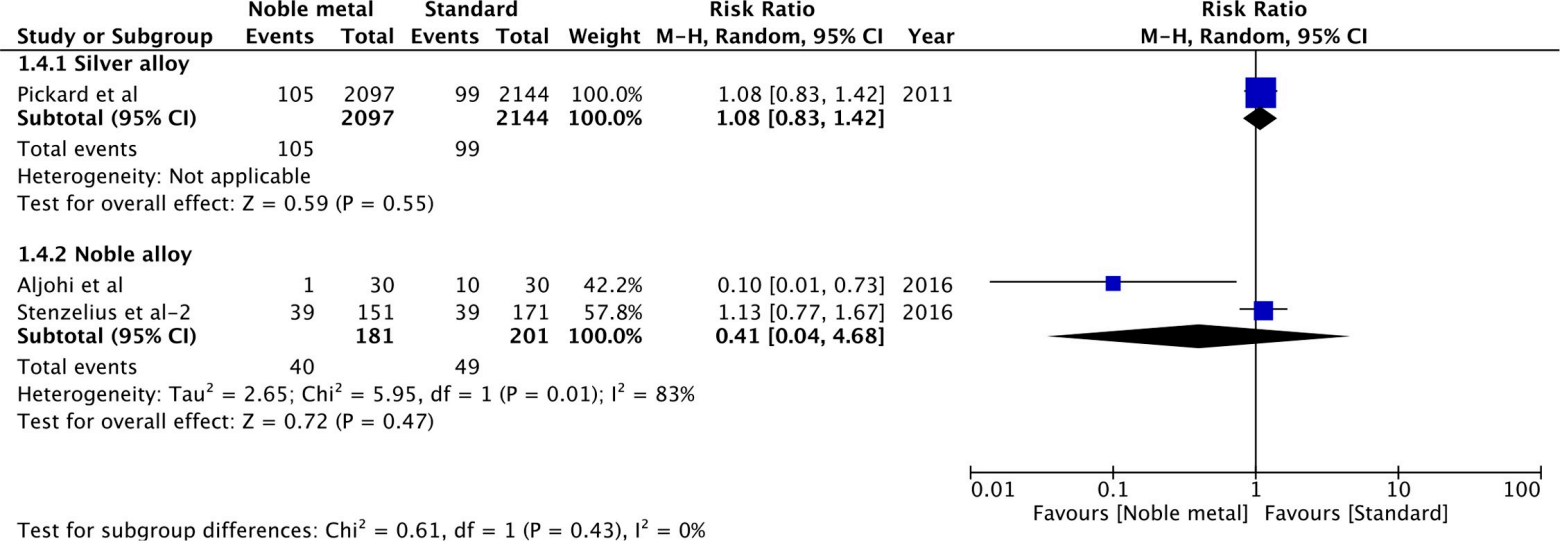
Forrest plot of symptomatic CAUTI.

Data for symptomatic CAUTI based on combined clinical and microbiological criteria was available from three studies [11,13,15] (Fig 5). In one large RCT [11] comparing silver alloy catheters vs standard catheters, there was no significant difference in symptomatic CAUTI between the two groups (RR 1.08, 95%CI 0.83–1.42, P = 0.55). Similarly, pooled data from two studies [13,15] did not demonstrate any significant difference between noble metal alloy catheters and standard catheters for the same outcome (RR 0.41, 95%CI 0.04–4.68, P = 0.47; I^2^ = 83%).

### Sensitivity analysis

On the exclusion of the second report of Verleyen et al [30] for bacteriuria with silver alloy catheter after >1week of catheterization, the results became statistically non-significant (RR 0.91, 95%CI 0.61–1.36, P = 0.64; I^2^ = 0%). The results of the two studies pooled for symptomatic CAUTI with noble metal alloy catheters were contrasting. Singularly, Aljohi et al [13] found a reduced risk of symptomatic CAUTI with noble metal alloy catheters while no such difference was seen by Stenzelius et al [15]. There were no other changes of effect direction on sensitivity analysis of the remaining variables.

### Risk of bias analysis

**Fig 6:**
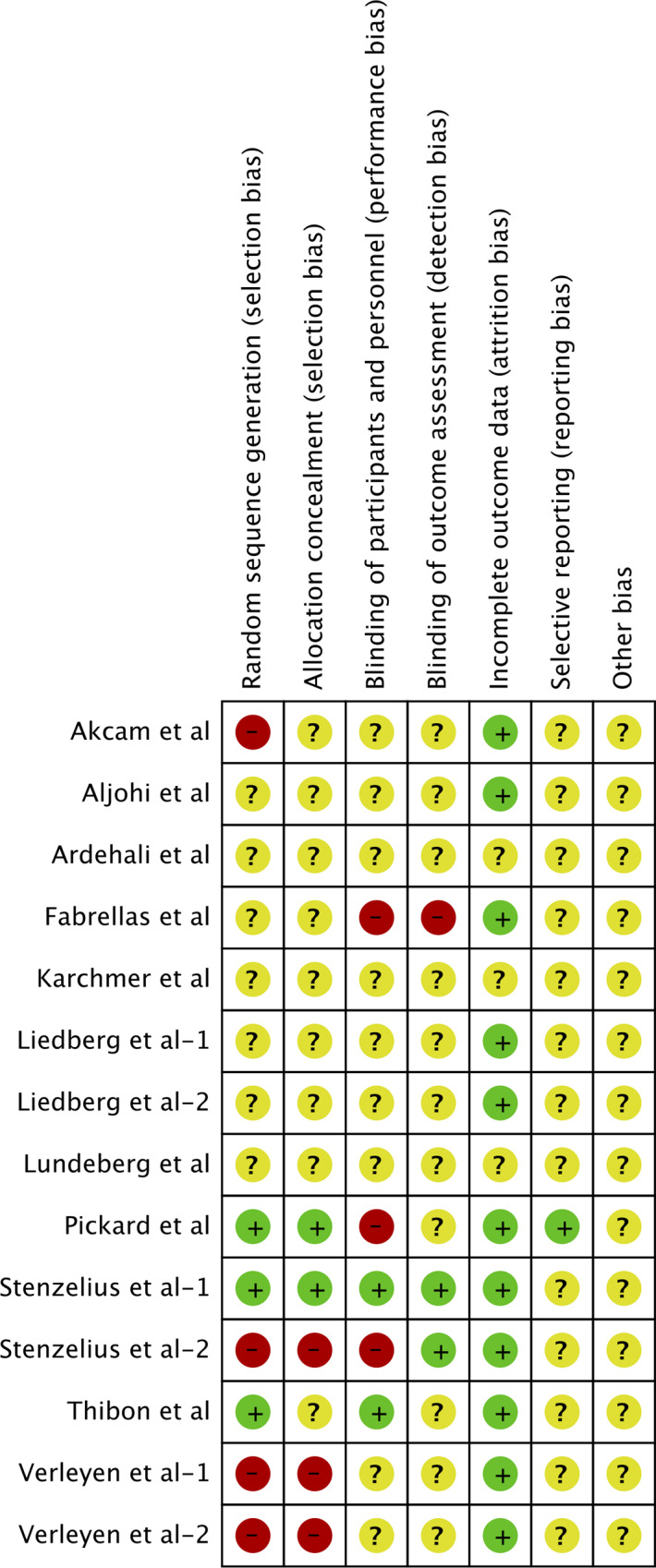
Risk of bias summary.

The risk of bias summary of included studies based on the authors' judgment is presented in Fig 6. Adequate method of randomization was described by three studies [11,31,32] and allocation concealment by two trials [11,32]. Blinding of participants and personnel [31,32] and blinding of outcome assessment [15,32] was appropriately clarified in two studies each.

## Discussion

CAUTIs contributed to a significant load of hospital-acquired infections worldwide. Several strategies to reduce CAUTIs have been proposed, which include limiting the use of catheter itself, minimizing the duration of catheterization, use of anti-septic methods during insertion and maintenance, use of disposable equipment, closed system catheterization and training of nursing staff and other personnel involved in patient care [5–7,33]. Another method involves coating the surface of the catheter with an anti-septic or antimicrobial like silver, noble metal alloy, nitrofurazone and chlorhexidine [7,8]. This technique is postulated to act directly against the primary mode of the pathogenesis of CAUTIs- the biofilm. The biofilm over an indwelling catheter consists of a reservoir of microbes and their extra-cellular products along with host-components. It offers a distinct survival advantage to pathogenic bacteria as the catheter surface does not have an inherent defense mechanism [35]. It is thought that coating the catheter surface with an agent acting against the colonizing microbes, may help reduce the incidence of CAUTIs.

Silver is a non-toxic noble metal and has been used as a bactericidal agent for burn wounds [9]. In 1979, Akiyama and Okamoto [36] devised the first urinary catheter coated with silver which was used in 102 patients. They found no incidence of bacteriuria (>10^5^ CFU/ml) in the study group while all 20 patients in the control group had bacteriuria in four days of catheterization. In the past decade, Bactiguard catheters (BIP Foley Catheter, Bactiguard AB, Stockholm, Sweden) have been introduced with an assertion of reduced CAUTIs [32]. In these catheters, in addition to silver, a very thin layer of other noble metals like gold and palladium are added to the surface coating. Since there have been no comparative studies of silver and noble metal alloy catheters to date, the results of these two devices were analyzed separately in our review.

In a systematic literature search, we found a total of seven studies [11,12,27–31] with eight reports comparing silver alloy catheters to standard catheters. Four reports [27–30] found a significant difference in the incidence of bacteriuria with silver-alloy catheters while the remaining four did not report any such difference. The overall results of our study indicate that silver-alloy catheters do reduce the incidence of bacteriuria for short-term catheterization (RR 0.63, 95%CI 0.44–0.90). Many of the included studies were however of a small sample size with none recruiting more than 100 participants in each group. Pickard et al [11], in the largest RCT included in the meta-analysis with 1785 patients in the study group and 1839 in the control group, found no significant reduction of bacteriuria with silver alloy catheters (RR 0.99, 95%CI 0.86–1.15). However, it is important to note that the median duration of catheterization in this trial was only 2 days (Inter-quartile range (IQR): 1–3 days) for both study and control groups which may have contributed to the non-significant result. On the other hand, while adequate methods of randomization and allocation concealment were employed in this study, the quality of four trials [27–30] reporting a significant difference in bacteriuria with silver alloy catheters was questionable. Similarly, on pooling data from four studies [13,14,32,33], we found a significant reduction of bacteriuria with the noble metal alloy catheters as compared to standard catheters. Our results concur with the previous meta-analysis of Lam et al [8] which reported similar results with both silver-alloy and noble metal alloy catheters. However, in comparison were able to add three more studies [13,14,33] evaluating noble alloy catheters and one more study [12] of silver alloy catheter to the previous review, thereby presenting updated evidence.

One important risk factor of CAUTI and bacteriuria is the duration of catheterization [32]. While it is hypothesized that noble metal coating may reduce the biofilm formation, no material has been found to date which eliminates bacterial colonization and biofilm formation; and bacteriuria may only be delayed with such catheters [35]. In a multicentric RCT, Bonfil et al [25] have compared silver-alloy catheters with standard catheters when used for a median time of 4 weeks. They found no significant difference between the two groups and did not recommend silver alloy-coated catheters for long-term use. While our study did not include trials utilizing catheters for more than 2 weeks, to test the validity of our results, we analyzed studies based on the duration of catheterization (more or less than 1 week). Our results indicated a significant reduction of bacteriuria with silver alloy catheters with more than one week of catheterization. However, the results should be interpreted with caution as they were from a small pool of studies with limited sample size and were not stable on sensitivity analysis. Since all studies using noble alloy catheters employed it for less than a week; it is not known if such catheters reduce bacteriuria with more than 1 week of catheterization.

The presence of bacteriuria, however, does not confirm CAUTI [15]. In patients with an indwelling catheter, a daily acquisition rate of bacteriuria is estimated to be 3–10%, with most cases of bacteriuria being asymptomatic and requiring no treatment [37]. A combined clinical and microbiological definition of CAUTI as suggested by the Centre for Disease Control and Prevention (CDC) is a better outcome variable rather than relying on bacteriuria alone for differences between noble metal-coated catheters and standard catheters [38]. Two large studies evaluating symptomatic CAUTI with silver alloy catheters presented conflicting results. While the results of Pickard et al [11] have already been discussed, Karchmer et al [10] in a large study on 27878 patients reported an estimated 21% reduction of risk of infection per 1000 patient-days and 32% reduction of risk of CAUTI per 100 silver-alloy catheters. The authors, however, used a combination of symptomatic UTI and asymptomatic bacteriuria to define CAUTI and data was not presented separately. For noble metal alloy catheters, pooled results of the two studies demonstrated a significant reduction of CAUTI with noble metal alloy catheters (RR 0.41, 95%CI 0.04–4.68). The results of the two studies [13,15] were conflicting with significant results seen only in the smaller trial of Aljohi et al [13] with 30 patients in each group.

Several limitations of our review need to be elaborated. Foremost, the quality of included studies was not high with only a few studies reporting adequate randomization, allocation concealment and blinding of personnel and outcome assessors. Secondly, there was wide methodological variability amongst studies with differences in the patient population studied, type of uncoated standard catheter, duration of catheterization, etc. Thirdly, the majority of included studies restricted the outcomes to the measurement of bacteriuria with only a few studies focusing on symptomatic CAUTI. Adverse events with the use of noble metal catheters were also not reported. Lastly, due to lack of data, a sub-group analysis based on variables like gender, use of antibiotics, method of specimen collection, etc could not be carried out. The strengths of our review include the updated evidence presented with the inclusion of four more studies from the last review. A sensitivity analysis was carried out to validate our results which were stable for the major outcome variable.

The involvement of nursing personnel in the prevention of hospital-acquired infection is continuously increasing, and the prevention of CAUTI is an integral component of this process. Since nurses have been independently managing urinary catheters in many hospitals worldwide, it is important that the selection and routine care of catheters is based on high-quality evidence. The aim of our review was to provide such level-1 evidence on the use of noble metal alloy catheters in adults. Our review indicates that bacteriuria may be reduced with the use of noble metal-coated catheters during short-term catheterization of adult, however, the quality of evidence is not high. It is not clear if these catheters reduce the risk of symptomatic CAUTI as evidence in this regard is conflicting. Further, homogenous trials may provide further clarity on this debatable topic.

## Supporting information

S1 Table Search strategy and results from PubMed database. (DOCX)

S1 Data (DOC)
