# Supplementary material for: Role of noble metal-coated catheters for short-term urinary catheterization of adults: a meta-analysis
Source: PLoS One. 2020 Jun 10;15(6):e0233215. doi: 10.1371/journal.pone.0233215 (PMC7286480; doi:10.1371/journal.pone.0233215)
Supplement: S1 Table — (DOCX) [file pone.0233215.s001.docx]

Supplementary table 1. Search strategy and results from PubMed database

| **Search number** | **Query** | **Search Details** | **Results** |
| --- | --- | --- | --- |
| 1 | (Silver oxide) AND (Foleys catheter) | (("disilver oxide"[Supplementary Concept] OR "disilver oxide"[All Fields]) OR "silver oxide"[All Fields]) AND ((("foley"[All Fields] OR "foley's"[All Fields]) OR "foleys"[All Fields]) AND ((("catheter's"[All Fields] OR "catheters"[MeSH Terms]) OR "catheters"[All Fields]) OR "catheter"[All Fields])) | 2 |
| 2 | (Silver alloy) AND (Foleys catheter) | (((("silver"[MeSH Terms] OR "silver"[All Fields]) OR "silvers"[All Fields]) OR "silvered"[All Fields]) AND ((((("alloy's"[All Fields] OR "alloyed"[All Fields]) OR "alloying"[All Fields]) OR "alloys"[MeSH Terms]) OR "alloys"[All Fields]) OR "alloy"[All Fields])) AND ((("foley"[All Fields] OR "foley's"[All Fields]) OR "foleys"[All Fields]) AND ((("catheter's"[All Fields] OR "catheters"[MeSH Terms]) OR "catheters"[All Fields]) OR "catheter"[All Fields])) | 14 |
| 3 | (Silver) AND (Foleys catheter) | ((("silver"[MeSH Terms] OR "silver"[All Fields]) OR "silvers"[All Fields]) OR "silvered"[All Fields]) AND ((("foley"[All Fields] OR "foley's"[All Fields]) OR "foleys"[All Fields]) AND ((("catheter's"[All Fields] OR "catheters"[MeSH Terms]) OR "catheters"[All Fields]) OR "catheter"[All Fields])) | 54 |
| 4 | ((Silver oxide) AND (urinary catheter)) AND (infection) | ((("disilver oxide"[Supplementary Concept] OR "disilver oxide"[All Fields]) OR "silver oxide"[All Fields]) AND (((("urinary catheters"[MeSH Terms] OR ("urinary"[All Fields] AND "catheters"[All Fields])) OR "urinary catheters"[All Fields]) OR ("urinary"[All Fields] AND "catheter"[All Fields])) OR "urinary catheter"[All Fields])) AND ((((((((((((((((((((("infect"[All Fields] OR "infectability"[All Fields]) OR "infectable"[All Fields]) OR "infectant"[All Fields]) OR "infectants"[All Fields]) OR "infected"[All Fields]) OR "infecteds"[All Fields]) OR "infectibility"[All Fields]) OR "infectible"[All Fields]) OR "infecting"[All Fields]) OR "infection"[MeSH Terms]) OR "infection"[All Fields]) OR "infections"[All Fields]) OR "infection's"[All Fields]) OR "infective"[All Fields]) OR "infectiveness"[All Fields]) OR "infectives"[All Fields]) OR "infectivities"[All Fields]) OR "infects"[All Fields]) OR "pathogenicity"[MeSH Subheading]) OR "pathogenicity"[All Fields]) OR "infectivity"[All Fields]) | 11 |
| 5 | ((Silver alloy) AND (urinary catheter)) AND (infection) | ((((("silver"[MeSH Terms] OR "silver"[All Fields]) OR "silvers"[All Fields]) OR "silvered"[All Fields]) AND ((((("alloy's"[All Fields] OR "alloyed"[All Fields]) OR "alloying"[All Fields]) OR "alloys"[MeSH Terms]) OR "alloys"[All Fields]) OR "alloy"[All Fields])) AND (((("urinary catheters"[MeSH Terms] OR ("urinary"[All Fields] AND "catheters"[All Fields])) OR "urinary catheters"[All Fields]) OR ("urinary"[All Fields] AND "catheter"[All Fields])) OR "urinary catheter"[All Fields])) AND ((((((((((((((((((((("infect"[All Fields] OR "infectability"[All Fields]) OR "infectable"[All Fields]) OR "infectant"[All Fields]) OR "infectants"[All Fields]) OR "infected"[All Fields]) OR "infecteds"[All Fields]) OR "infectibility"[All Fields]) OR "infectible"[All Fields]) OR "infecting"[All Fields]) OR "infection"[MeSH Terms]) OR "infection"[All Fields]) OR "infections"[All Fields]) OR "infection's"[All Fields]) OR "infective"[All Fields]) OR "infectiveness"[All Fields]) OR "infectives"[All Fields]) OR "infectivities"[All Fields]) OR "infects"[All Fields]) OR "pathogenicity"[MeSH Subheading]) OR "pathogenicity"[All Fields]) OR "infectivity"[All Fields]) | 60 |
| 6 | ((Silver) AND (urinary catheter)) AND (infection) | (((("silver"[MeSH Terms] OR "silver"[All Fields]) OR "silvers"[All Fields]) OR "silvered"[All Fields]) AND (((("urinary catheters"[MeSH Terms] OR ("urinary"[All Fields] AND "catheters"[All Fields])) OR "urinary catheters"[All Fields]) OR ("urinary"[All Fields] AND "catheter"[All Fields])) OR "urinary catheter"[All Fields])) AND ((((((((((((((((((((("infect"[All Fields] OR "infectability"[All Fields]) OR "infectable"[All Fields]) OR "infectant"[All Fields]) OR "infectants"[All Fields]) OR "infected"[All Fields]) OR "infecteds"[All Fields]) OR "infectibility"[All Fields]) OR "infectible"[All Fields]) OR "infecting"[All Fields]) OR "infection"[MeSH Terms]) OR "infection"[All Fields]) OR "infections"[All Fields]) OR "infection's"[All Fields]) OR "infective"[All Fields]) OR "infectiveness"[All Fields]) OR "infectives"[All Fields]) OR "infectivities"[All Fields]) OR "infects"[All Fields]) OR "pathogenicity"[MeSH Subheading]) OR "pathogenicity"[All Fields]) OR "infectivity"[All Fields]) | 176 |
| 7 | (Noble metal catheter) AND (infection) | (("noble"[All Fields] OR "nobles"[All Fields]) AND ((((((((((((((((((((((((((("metal's"[All Fields] OR "metalate"[All Fields]) OR "metalated"[All Fields]) OR "metalates"[All Fields]) OR "metalating"[All Fields]) OR "metalation"[All Fields]) OR "metalations"[All Fields]) OR "metalative"[All Fields]) OR "metalic"[All Fields]) OR "metalization"[All Fields]) OR "metalized"[All Fields]) OR "metallate"[All Fields]) OR "metallated"[All Fields]) OR "metallates"[All Fields]) OR "metallation"[All Fields]) OR "metallations"[All Fields]) OR "metallic"[All Fields]) OR "metallically"[All Fields]) OR "metallicities"[All Fields]) OR "metallicity"[All Fields]) OR "metallics"[All Fields]) OR "metallization"[All Fields]) OR "metallizations"[All Fields]) OR "metallize"[All Fields]) OR "metallized"[All Fields]) OR "metals"[MeSH Terms]) OR "metals"[All Fields]) OR "metal"[All Fields]) AND ((("catheter's"[All Fields] OR "catheters"[MeSH Terms]) OR "catheters"[All Fields]) OR "catheter"[All Fields])) AND ((((((((((((((((((((("infect"[All Fields] OR "infectability"[All Fields]) OR "infectable"[All Fields]) OR "infectant"[All Fields]) OR "infectants"[All Fields]) OR "infected"[All Fields]) OR "infecteds"[All Fields]) OR "infectibility"[All Fields]) OR "infectible"[All Fields]) OR "infecting"[All Fields]) OR "infection"[MeSH Terms]) OR "infection"[All Fields]) OR "infections"[All Fields]) OR "infection's"[All Fields]) OR "infective"[All Fields]) OR "infectiveness"[All Fields]) OR "infectives"[All Fields]) OR "infectivities"[All Fields]) OR "infects"[All Fields]) OR "pathogenicity"[MeSH Subheading]) OR "pathogenicity"[All Fields]) OR "infectivity"[All Fields]) | 11 |
| 8 | (Noble metal catheter) AND (urinary tract infection) | (("noble"[All Fields] OR "nobles"[All Fields]) AND ((((((((((((((((((((((((((("metal's"[All Fields] OR "metalate"[All Fields]) OR "metalated"[All Fields]) OR "metalates"[All Fields]) OR "metalating"[All Fields]) OR "metalation"[All Fields]) OR "metalations"[All Fields]) OR "metalative"[All Fields]) OR "metalic"[All Fields]) OR "metalization"[All Fields]) OR "metalized"[All Fields]) OR "metallate"[All Fields]) OR "metallated"[All Fields]) OR "metallates"[All Fields]) OR "metallation"[All Fields]) OR "metallations"[All Fields]) OR "metallic"[All Fields]) OR "metallically"[All Fields]) OR "metallicities"[All Fields]) OR "metallicity"[All Fields]) OR "metallics"[All Fields]) OR "metallization"[All Fields]) OR "metallizations"[All Fields]) OR "metallize"[All Fields]) OR "metallized"[All Fields]) OR "metals"[MeSH Terms]) OR "metals"[All Fields]) OR "metal"[All Fields]) AND ((("catheter's"[All Fields] OR "catheters"[MeSH Terms]) OR "catheters"[All Fields]) OR "catheter"[All Fields])) AND (((("urinary tract infections"[MeSH Terms] OR (("urinary"[All Fields] AND "tract"[All Fields]) AND "infections"[All Fields])) OR "urinary tract infections"[All Fields]) OR (("urinary"[All Fields] AND "tract"[All Fields]) AND "infection"[All Fields])) OR "urinary tract infection"[All Fields]) | 8 |
| 9 | (Silver alloy) AND (urinary tract infection) | (((("silver"[MeSH Terms] OR "silver"[All Fields]) OR "silvers"[All Fields]) OR "silvered"[All Fields]) AND ((((("alloy's"[All Fields] OR "alloyed"[All Fields]) OR "alloying"[All Fields]) OR "alloys"[MeSH Terms]) OR "alloys"[All Fields]) OR "alloy"[All Fields])) AND (((("urinary tract infections"[MeSH Terms] OR (("urinary"[All Fields] AND "tract"[All Fields]) AND "infections"[All Fields])) OR "urinary tract infections"[All Fields]) OR (("urinary"[All Fields] AND "tract"[All Fields]) AND "infection"[All Fields])) OR "urinary tract infection"[All Fields]) | 59 |
| 10 | (SIlver) AND (urinary tract infection) | ((("silver"[MeSH Terms] OR "silver"[All Fields]) OR "silvers"[All Fields]) OR "silvered"[All Fields]) AND (((("urinary tract infections"[MeSH Terms] OR (("urinary"[All Fields] AND "tract"[All Fields]) AND "infections"[All Fields])) OR "urinary tract infections"[All Fields]) OR (("urinary"[All Fields] AND "tract"[All Fields]) AND "infection"[All Fields])) OR "urinary tract infection"[All Fields]) | 248 |
| 11 | (Silver oxide) AND (urinary catheter) | (("disilver oxide"[Supplementary Concept] OR "disilver oxide"[All Fields]) OR "silver oxide"[All Fields]) AND (((("urinary catheters"[MeSH Terms] OR ("urinary"[All Fields] AND "catheters"[All Fields])) OR "urinary catheters"[All Fields]) OR ("urinary"[All Fields] AND "catheter"[All Fields])) OR "urinary catheter"[All Fields]) | 13 |
| 12 | (Silver alloy) AND (urinary catheter) | (((("silver"[MeSH Terms] OR "silver"[All Fields]) OR "silvers"[All Fields]) OR "silvered"[All Fields]) AND ((((("alloy's"[All Fields] OR "alloyed"[All Fields]) OR "alloying"[All Fields]) OR "alloys"[MeSH Terms]) OR "alloys"[All Fields]) OR "alloy"[All Fields])) AND (((("urinary catheters"[MeSH Terms] OR ("urinary"[All Fields] AND "catheters"[All Fields])) OR "urinary catheters"[All Fields]) OR ("urinary"[All Fields] AND "catheter"[All Fields])) OR "urinary catheter"[All Fields]) | 62 |
| 13 | (Silver) AND (urinary catheter) | ((("silver"[MeSH Terms] OR "silver"[All Fields]) OR "silvers"[All Fields]) OR "silvered"[All Fields]) AND (((("urinary catheters"[MeSH Terms] OR ("urinary"[All Fields] AND "catheters"[All Fields])) OR "urinary catheters"[All Fields]) OR ("urinary"[All Fields] AND "catheter"[All Fields])) OR "urinary catheter"[All Fields]) | 200 |
